# Inadequate knowledge on appropriate antibiotics use among clients in the Moshi municipality Northern Tanzania

**DOI:** 10.1371/journal.pone.0239388

**Published:** 2020-09-24

**Authors:** Erick Alexander Mboya, Matthew Lee Davies, Pius Gerald Horumpende, James Samwel Ngocho

**Affiliations:** 1 Department of Epidemiology and Biostatistics, Muhimbili University of Health and Allied Sciences, Dar Es Salaam, Tanzania; 2 Population Health Sciences Institute, Newcastle University, Newcastle Upon Tyne, United Kingdom; 3 Infectious Diseases Institute, Military College of Medical Sciences, Lugalo, Dar Es Salaam, Tanzania; 4 Kilimanjaro Clinical Research Institute, Kilimanjaro, Tanzania; 5 Institute of Public Health, Kilimanjaro Christian Medical University College, Kilimanjaro, Tanzania; University of Toronto, Rotman School, CANADA

## Abstract

**Background:**

Poor knowledge concerning appropriate antibiotic use significantly influences the misuse of antibiotics within the community, especially in developing countries where there are weaker health systems to regulate antibiotic dispensing. Antibiotic misuse leads to antibiotic resistance. This study assessed knowledge of appropriate antibiotic use among buyers in the Moshi municipality, Northern Tanzania.

**Methods:**

We conducted a cross-sectional study in Moshi municipality between April and May 2017. Adults who bought antibiotics at drug outlets were invited to participate in the study. An exit interview was conducted with participants to collect their demographics and assess their knowledge concerning appropriate use of antibiotics. A logistic regression model was performed to determine factors associated with correct knowledge concerning antibiotic use.

**Results:**

A total of 152 adults with a median age of 30.5 (IQR 25–42) years, were enrolled in the study. Slightly over half (n = 89, 58.6%), responded that they should stop antibiotics after finishing the dose as directed. Half (n = 77, 50.7%) thought that it was acceptable to share antibiotics with other individuals and over half of respondents (n = 95, 65.1%) thought that they should request the same antibiotics if they had used them to treat a similar illness in the past. Only 38 (25%) had adequate knowledge about the use of antibiotics. Sore throat and flu were respectively identified by 62.5% and 46.1% of respondents as conditions that can be treated with antibiotics. Higher levels of education (aOR = 4.11 95%CI = 1.44–11.71) and having health insurance (aOR = 9.05 95%CI = 3.35–24.45) were associated with better levels of knowledge concerning antibiotic use in various illnesses.

**Conclusion:**

There is inadequate knowledge concerning the indications for antibiotics and their appropriate usage. Health promotion campaigns are needed to educate the population about appropriate antibiotic use and reduce their irrational use.

## Introduction

The irrational use of antibiotics refers to their inappropriate use, for instance to treat viral illness or for the wrong duration or dose [1, 2]. Development of resistance to the commonly available antibiotics is a direct consequence of their irrational use [3]. It is estimated that over 80% of all antibiotics, in most countries, are used in the community, where monitoring of their rational use is challenging [4].

One of the major contributors to irrational antibiotic use is poor knowledge within the community on their correct usage [5]. For example, antibiotics are often believed to be “wonder drugs” with the ability to cure any ailment. Such attitudes greatly influence the irrational use of antibiotics in the community [3, 6]. Lack of knowledge about the activity of antibiotics on bacteria and in the inactivity of antibiotics on other pathogens (such as viruses, fungi and parasites), and about the emergence of antibiotic resistance in case of inappropriate antibiotic use, also influence irrational use [7, 8].

The situation is much worse in developing countries owing to weak health systems, which lack mechanisms to monitor dispensing, despite appropriate policies, rules and regulations often being in place on antibiotic prescriptions and dispensing [1, 7, 9]. Studies in Africa have shown irrational usage accounting for 75% to 100% of antibiotic use in the community [5, 10–12]. Antibiotics are commonly and irrationally used to treat non-bacterial illnesses, especially upper respiratory tract infections (URTI) and diarrhoea [5, 13, 14].

In Tanzania, antibiotics are classified as prescription only medicines where only the few antibiotics allowed to be sold or dispensed by the part II shops and a wider range allowed to be sold and dispensed from part I shops (Pharmacies). The selling of drugs in the retail outlets is regulated by the Pharmacy Council (PC) under the Ministry of Health Community Development, Gender, Elderly and Children (MOHCDGEC) [15]. Dispensers often abandon correct dispensing practices for financial reasons [16]. Even with regular reminders and continuing education provided to the dispensers by regulatory bodies, little has changed [17, 18]. Poor dispensing practices escalate the burden of antibiotic resistance, consequently increasing the cost of treatment and leading to poor treatment outcomes [14, 19].

Interventions to provide education to the community have proven to be successful in reducing overall antibiotic usage and in slowing the increase in antibiotic resistance in some developed countries [8, 20]. However, interventions that address the knowledge gaps on antibiotic use are often not well established, especially in low- and middle-income countries (LMIC). Data on the level of knowledge on appropriate antibiotic use, which is essential for formulating effective interventions, is scarce in these settings, Tanzania inclusive. Here, we report on the level of knowledge concerning appropriate antibiotic use among clients at community drug outlets in Moshi municipality, Northern Tanzania.

## Materials and methods

### Design and settings

This was an exit interview, cross-sectional study among antibiotic buyers from drug outlets in Moshi Municipality conducted between April and May 2017. Moshi municipality is one of seven administrative councils of the Kilimanjaro region, Northern- eastern Tanzania. In 2017, it had an estimated 201,150 residents [21]. It had a total of 20 pharmacies and 37 Accredited Drug Dispensing Outlets (ADDOs) operating during the time of this study. At the pharmacies (Part I shops), pharmaceutical technicians and pharmacy assistants operate under the direct supervision of a registered pharmacist. ADDOs (part II shops) may have a pharmaceutical technician or assistant but, if not, they are required to have an accredited drug dispenser who has completed a five-week training course. A total of 12 outlets were included in this study, 5 were pharmacies and 7 were ADDOs, as previously described [5].

### Population

The study population were adults who bought antibiotics from the community outlets during the study period.

### Sampling and sample size

The minimum sample size was calculated using the formula for a single population proportion, as previously described [5]. A simple random sampling method was used in selecting the drug outlets. Individuals who bought antibiotics from the selected drug outlets during the study period and who met the inclusion criteria were invited to participate in the study.

### Procedure

After an individual purchased an antibiotic, the drug dispenser informed the clients of the ongoing study and directed them to the investigator, who was outside the store. The investigator introduced himself to the clients, briefly introduced the study objectives and asked for a signed consent to participate. After consenting, an exit interview using a questionnaire was conducted outside the drug outlet. The interviews were conducted in Swahili by the investigator and took about 30 minutes to complete.

### Measures

In addition to general and demographic characteristics like age, sex, residence, and marital status, other variables were captured such as employment, and health-insurance status. Knowledge concerning the use of antibiotics was assessed using questions adapted from a validated WHO questionnaire used in a multi-country survey [22].

Knowledge about the use of antibiotics was assessed using three questions:

1. When do you think you should stop antibiotics once you have begun treatment?
2. Is it okay to use antibiotics given to someone else—for example a friend or a family member—as long they were used for treating the same illness?
3. If you are sick, is it okay to buy, or to request the same antibiotics from a doctor, if they helped you get better when you had same symptoms previously?

Each question’s response was analyzed individually as a component of knowledge concerning the use of antibiotics.

In addition, knowledge about using antibiotics in treating different common conditions, both bacterial and non-bacterial, was assessed. Participants were asked a question “do you think antibiotics can be used to treat malaria?”. Questions further asked whether antibiotics can be used to treat other conditions such that sore throat, flu, diarrhea, headache, body aches, fever, infected skin wound, and urinary tract infection (UTI). Overall knowledge in using antibiotics in different conditions was assessed as being good if participants answered correctly at least four out of the nine conditions.

### Data analysis

Data were entered in a database and cleaned before checked for completeness. Data were then analyzed using the Statistical Package for Social Sciences (SPSS) version 25 (IBM USA). The age profile of participants was summarized by calculating the median age and the interquartile range in years. Categorical variables including the general characteristics of participants (sex, marital status, health insurance status, education level, employment and prior hospital attendance), knowledge about the use of antibiotics and responses on questions about the use of antibiotics in different conditions, were summarized using frequencies and percentages.

For knowledge about antibiotic use, measures of association with individual demographic characteristics were calculated using cross tabulation and a binary logistic regression model. Odds ratios were calculated and a p value of < 0.05 was the cut off point for significance. A multivariate logistic regression model was used to assess the role of each demographic characteristic in independently accounting for buyer knowledge. For each individual condition mentioned above, measures of association using univariate binary logistic regression and multivariate logistic regression model were used to predict the association with individual characteristics such as age, gender, education level, having formal employment and health insurance where a p value of <0.05 was used as a cut-off point for significance.

### Ethical considerations

Ethical clearance for this study was granted by the Kilimanjaro Christian Medical University College Research ethics committee. Permission to conduct the study was obtained from the drug outlets owners and written informed consents were obtained from the participants prior to interview.

## Results

A total of 152 adults who bought antibiotics in the selected drug outlets during the study period were enrolled in the study. The median age of participants was 30.5 (IQR = 25–42) years. Females accounted for 61.8% of participants. Almost half of the participants, 48%, had secondary education. Only 15.1% of the participants had formal employment in the last 12 months, only about a quarter (27.6%) had health insurance and only on quarter of participants had prescription for the antibiotic they bought. The outlet types contributed almost equally almost equal number of participants in this study (Table 1).

**Table 1:** General characteristics of the study participants and outlet types (N = 152).

Characteristic	N	%
**Sex**		
Male	58	38.2
Female	94	61.8
**Age group (years)**		
Median age = 30.5 (IQR 25–42)		
18–25	42	27.6
26–35	59	38.8
36–45	24	15.8
46–55	12	7.9
>55	15	9.9
**Marital status**		
Married	91	59.9
Not Married	61	40.1
**Education level**		
Primary	44	28.9
Secondary	73	48.0
Diploma/ Certificate	19	12.5
Bachelor/ Master’s Degree	16	10.5
**Health Insurance**		
Yes	42	27.6
No	110	72.4
**Formal Employment**		
Yes	23	15.1
No	129	84.9
**Did you go to the hospital prior coming here?**		
Yes	60	39.5
No	92	60.5
**Do you have a prescription for this antibiotic?**		
Yes	36	23.7
No	116	76.3
**Outlet type**		
Part I shops (Pharmacy)	71	46.7
Part II shops (ADDOs)	81	53.3

### Knowledge on how and when to take or stop taking antibiotics

Slightly over half of the participants, 89 (58.6%), responded that they should stop antibiotics after finishing the dose as directed, while more than a quarter of participants, 58 (38.2%), thought that antibiotics should be stopped when they felt better. About half of respondents, 77 (50.7%), thought that they should use antibiotics from a friend or a family member, if they had used them to treat the same or a similar illness previously. A majority of participants, 95 (65.1%), thought that they should request a specific antibiotic, if they had used it previously in treating the same symptoms/disease previously. Overall, 25% of participants had good knowledge concerning the use of antibiotics (Table 2).

**Table 2:** Responses on knowledge around the use of antibiotics (N = 152).

Variable	n (%)
**Do you think it is good to use the same antibiotic if a friend or family member used to treat same symptom or disease before?**	
Yes	77 (50.7%)
No	75 (49.3%)
**Do you think it is good to ask/ request the same antibiotic if it helped you treat the same symptoms/ disease previously?**	
Yes	99 (65.1%)
No	53 (34.9%)
**When do you think you should stop taking antibiotics once you have started treatment?**	
When I finish the dose as directed	89 (58.6%)
When I feel better	58 (38.2%)
I don’t know	05 (03.3%)
**Overall knowledge around the use of antibiotics**	
**Good**	38 (25%)
**Poor**	114 (75%)

### Knowledge concerning the use of antibiotics and individual characteristics

A larger proportion of females compared to males disagreed when asked if it is good to request antibiotics if they helped to treat the same symptoms/disease previously: (38.3% and 29.3% respectively). Knowledge about sharing of and when to stop antibiotics was comparable between the sexes. Larger proportions of individuals with university or college education, formal employment, health insurance and those who attended to the hospital prior coming to the outlet disagreed on when to stopping antibiotics when they felt better, on sharing of antibiotics with a friend or family member and on requesting of antibiotics from a doctor to treat same symptoms as previously used for, compared to their counterparts (Table 3). Among those who had prescriptions, a larger proportion disagreed on the request of the same antibiotics if it helped treating the same symptoms previously and on the sharing of the antibiotics with friends and family for same symptoms compared to the individuals who did not have prescription. A larger proportion of individuals with prescription disagreed on stopping antibiotics when they felt better compared to those that did not have prescriptions (Table 3)

**Table 3:** Correct responses (Good knowledge) around antibiotics use with participants characteristics.

Variable	When do you think you should stop taking antibiotics once you have started treatment?	Do you think it is good to use the same antibiotic if a friend or family member used it to treat same symptom or disease before?	Do you think it is good to ask/ request the same antibiotic if it helped you treat the same symptoms/ disease previously?	
	n (%)	n (%)	n (%)	Total
**Sex**				
Male	33 (56.7)	29 (50.0)	17 (29.3)	58
Female	56 (59.6)	46 (48.9)	36 (38.3)	94
**Age group (years)**				
**<35**	60 (59.4)	51 (50.5)	36 (35.6)	101
**36–55**	21 (58.3)	15 (41.7)	12 (33.3)	36
**>55**	08 (53.3)	09 (60.0)	05 (33.3)	15
**Marital status**				
Married	54 (59.3)	44 (48.4)	30 (33.0)	91
Not Married	35 (57.4)	31 (50.8)	23 (37.7)	61
**College/ University Education**				
**Yes**	23 (65.7)	24 (68.6)	17 (48.6)	35
**No**	66 (56.4)	51 (43.6)	36 (30.8)	117
**Formal employment**				
**Yes**	16 (69.6)	17 (73.9)	13 (56.5)	23
**No**	73 (56.6)	58 (45.0)	40 (31.0)	129
**Health Insurance**				
**Yes**	27 (64.3)	28 (66.7)	18 (42.9)	42
**No**	62 (56.4)	47 (42.7)	35 (31.8)	110
**Did you go to the hospital prior coming here?**				
Yes	41 (68.3)	35 (58.3)	27 (45.0)	60
No	48 (52.2)	40 (43.5)	26 (28.3)	92
**Do you have a prescription for this antibiotic?**				
Yes	23 (63.9)	24 (66.7)	21 (58.3)	36
No	66 (56.9)	51 (44.0)	32 (27.6)	116

### Conditions that can be treated with antibiotics

**Fig 1:**
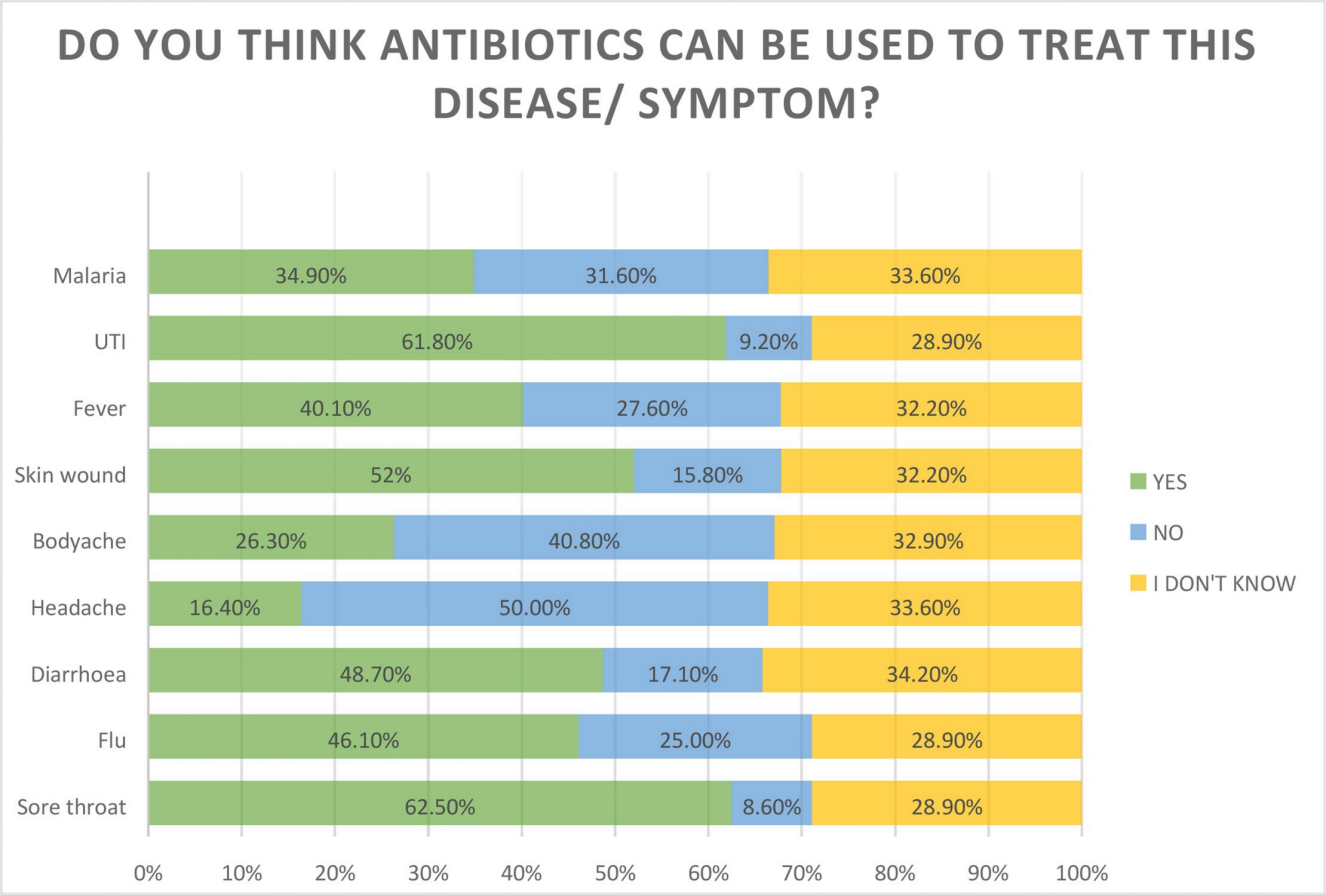
Responses on conditions that can be treated with antibiotic.

Common conditions that were identified as treatable with antibiotics were sore throat (62.5%), flu (46.1%), fever (40.1%) and malaria (34.9%). Almost half of all respondents, 48%, agreed that antibiotics can be used to treat diarrhoea, and only 17.1% disagreed with this statement. Malaria was identified by 34.9% of participants as one of the diseases that can be treated with antibiotics. UTI and infected skin wounds were identified by most respondents, 61.8% and 52% respectively, as conditions that can be treated with antibiotics (Fig 1). Overall, 51 participants (33.6%), had appropriate knowledge on antibiotic use in treating different conditions.

### Factors associated with appropriate knowledge on antibiotic use

Good knowledge on antibiotic use in various illnesses was significantly associated with having a higher level of education and health insurance. Individuals with health insurance were nine times more likely to have overall good knowledge on the appropriate use of antibiotics in various illnesses than the individuals with no health insurance (aOR = 9.05, 95% CI = 3.35–24.45). Individuals with college or university education were four times more likely to have good knowledge on antibiotic use in various illnesses compared to their counterparts (aOR = 4.11 95%CI = 1.44–11.71). Other factors such as sex, age, formal employment, hospital visit prior to coming to the outlet and having a prescription for the bought antibiotic were not significantly associated with good knowledge on antibiotic use (Table 4).

**Table 4:** Factors associated with good knowledge on antibiotics usage in different common conditions.

Variable	cOR (95%CI)	p-value	aOR (95%CI)	p-value
**Sex**				
Male	1.07(0.54–2.14)	0.849	0.77 (0.32–1.80)	0.542
Female				
**Age groups (years)**				
0–35	0.56(0.19–1.66)	0.292	1.02 (0.24–4.39)	0.976
36–55	0.50(0.15–1.73)	0.276	0.67 (0.14–3.13)	0.606
>55				
**Education level**				
College/ University	4.35(1.97–9.62)	<0.001	4.11 (1.44–11.71)	0.008
No college/ university				
**Formal employment**				
Yes	2.04(0.83–5.01)	0.120	0.41 (0.11–1.47)	0.169
No				
**Health insurance**				
Yes	7.57 (3.44–16.66)	<0.001	9.05 (3.35–24.45)	<0.001
No				
**Prior hospital visit**				
Yes				
No	0.67 (0.33–1.36)	0.272	2.19 (0.78–6.16)	0.136
**Do you have a prescription for this antibiotic?**				
Yes	0.98 (0.46–2.24)	0.975	1.789 (0.56–5.67)	0.323
No				

*Adjusted for Education level, employment status, health insurance, visiting the hospital prior coming to the outlet, having a prescription. gender and age.

## Discussion

This cross-sectional study assessed the level of knowledge concerning appropriate antibiotic use among clients in the community drug outlets within the Moshi municipality, Northern Tanzania. We found that three quarters (75%) of individuals in the municipality have poor knowledge of appropriate antibiotic use (when to stop, requesting antibiotics and sharing of antibiotics). This is an unacceptably high level of poor knowledge on the use of antibiotics in this era where WHO declared antimicrobial resistance a world crisis since 2014 [23]. We also found that two thirds (66.4%) of the individuals do not know what antibiotics are used for. Levels of knowledge, in this regard, were significantly lower in those with less education and those without health insurance. Deliberate efforts to provide the public with antibiotic knowledge in terms of public education cannot be overemphasized.

With regard to knowledge about appropriate antibiotic use, 41.4% of participants demonstrated poor knowledge concerning when they should stop taking antibiotics once they had started treatment. These respondents either did not know when they should stop treatment or thought that they should cease antibiotics when they felt better. In a study carried out in 2017 in a similar setting, 81.8% of the general population disagreed with stopping antibiotics on symptoms improving compared to a lower proportion, 58.6%, in this study [24]. This can be due to the difference in the population involved in the two studies, as the general population may have a better knowledge compared to those accessing antibiotics in these outlets where almost 90% of antibiotics are irrational. A relatively smaller sample size in this study may also be the reason for this difference. Asamoah et al, working in Ghana, found that only a small proportion of individuals (15%) actually finish their antibiotics as directed [25]. Hence, even when there might be good knowledge, there can still be a gap in practice, which risks emerging of resistance to the commonly available antibiotics [3, 25]. In Tanzania, it has also been shown that most dispensers do not provide instructions to clients on how to use antibiotics properly [13]. There is, therefore, a significant scope for dispensers to improve the knowledge of their clients on antibiotic usage, for one part, the importance of finishing the course of antibiotics.

Another aspect of knowledge assessed was regarding requesting the same antibiotics from a doctor when the client had used it previously to treat a similar illness or symptoms. Sixty five percent of participants agreed that this was appropriate while this is wrong. Requests for antibiotics pressurises clinicians and dispensers, and can influence them to prescribe antibiotics incorrectly [26]. Kajeguka et al found that, among the general population in a similar setting, 58.9% did not expect an antibiotic each time they visited a doctor, compared to 34.9% of buyers in this study [24]. In developed countries, requesting antibiotics from doctors has been reported to be over 70%, with upper respiratory tract infections being one of the main reasons [27]. Overt and non-overt pressure has been reported to influence prescribers and dispensers to prescribe and dispense antibiotics unnecessarily [16, 26].

The final aspect on knowledge assessment was in relation to sharing antibiotics. In this study, 50.6% of participants thought it is acceptable to share the same antibiotics with a family member or friend if they used it to treat the same illness or symptoms. In another study, conducted in a similar setting, only 16.8% approved of sharing antibiotics [24]. Poor knowledge on sharing antibiotics in Moshi municipality was twice as high compared to that reported in a survey of 12 countries conducted by the WHO in 2015. In the WHO study, only 25% of respondents agreed that it was acceptable to use the same antibiotics given to a friend or family member as long as they were used to treat the same illness [22]. Again, differences in the populations included in the two studies may explain the variation in the responses, as individuals who uses antibiotics irrationally are more likely to have poor knowledge and are acquiring antibiotics through the community outlets where they are easily accessible and poorly monitored. Also, it is worth noting that sharing antibiotics can represent a reason for not finishing antibiotics in the first place.

Overall, only a quarter of participants had good knowledge on the use of antibiotics. The consequences of this include unnecessary prescriptions from doctors, sharing of antibiotics and not completing antibiotics prescribed as directed [5]. These factors expose bacteria to suboptimal doses of antibiotics and facilitate emergence of resistance to the commonly available antibiotics [3, 7].

We also assessed knowledge about what antibiotics are used for. Overall, we found poor knowledge among buyers on what antibiotics are used for. To start with, most participants thought that a sore throat can be treated with antibiotics. In addition, 46.1% of participants thought that flu can be treated with antibiotics, while only slightly over a quarter, 28.6%, disagreed with the statement. These results should not come as a surprise given that URTIs are consistently the leading complaint for which antibiotics are sought in most parts of the world, Tanzania included [5, 27–29]. Findings from Namibia were similar, where 44% and 49% of individuals respectively agreed that colds and flu could be treated with antibiotics [30]. A slightly lower prevalence has been reported in the southern America region where on average 33% of URTI are treated with antibiotics. However, much variations have been reported within that region where up to 94% of URTI are treated with antibiotics in Bolivia and much lower levels of URTI, 21% are treated with antibiotics in Uruguay [31].

Almost half of all participants (48%) agreed that non bloody diarrhoea should be treated with antibiotics. The WHO found similar results for knowledge about antibiotic use in treating diarrhoea in a survey of 12 countries worldwide, in which 43% of the respondents agreed with using antibiotics to treat diarrhoea [22]. Bojajil et al reported almost similar but slightly lower levels of antibiotic use in Mexico, where about 32% of diarrhoea episode were treated with antibiotics in the households. Similarly, in the middle east and central Asia, poor knowledge on antibiotics use has been reported and subsequently diarrhoea is among the leading indication for antibiotic use [32, 33].

Studies carried out in Tanzania have shown that URTIs and diarrhoea are common reasons for irrational antibiotic use. Further, in the similar setting, patients with acute watery diarrhoea and URTI have also been documented to be highly inappropriately prescribed with antibiotics by clinicians as well. [5, 34]. The situation, with regard to poor antibiotic knowledge to antibiotic use to treat URTIs and diarrhoea, is similar in many places worldwide, as studies in Ethiopia, India, Vietnam, USA, China have demonstrated [10, 35–39].

Overall, only one third (33.6%) of participants had good knowledge about the illnesses that can be treated with antibiotics. These concerning misconceptions translate into poor practise as most individuals seek antibiotics to treat non-bacterial illnesses [5].

Factors that had a significant association with good understanding of the conditions that warrant antibiotic use included having health insurance and higher education level. Similar findings have been reported by Parimi et al and Nepal et al where individuals with higher levels of education were reported to have better knowledge on appropriate antibiotic use compared to those with lower levels of education [40]. Again, concordant findings have been reported by Parimi et al for health insurance, where individuals with access to health insurance were more likely to have adequate knowledge on antibiotic use than their counterparts without [41]. This might be explained by the fact that the individuals who can access health insurance are often of higher socio-economic status and higher levels of education.

### Strength and limitations

A strength of this study is that it assessed the knowledge of those individuals purchasing antibiotics in community outlets. However, caution in interpreting the study’s results is required, since the method of data collection (exit interviews) might have introduced information bias. For instance, a potential source of information bias might have occurred where the dispenser coached the customer on the knowledge and use of use antibiotics before directing them to the interviewer. An additional limitation is that the study did not follow up on the actual practice. This might have differed from the buyer’s responses to the questions. This is an avenue for further research as it was beyond the scope of this study.

## Conclusion and recommendation

The study found inadequate knowledge on how, when and what antibiotics should be used for among antibiotic clients in the Moshi municipality. To combat irrational antibiotic use community health promotion campaigns that carry specific messages addressing local knowledge gaps in the local population should be formulated and implemented. The task is possible through one of the technical working groups on National Action Plan (NAP) in Tanzania’s Ministry of Health, Community Development, Gender, Elderly and Children. This will result in effective reduction of irrational antibiotic use and contribute to slowing the emergence and spread of resistance to commonly available antibiotics.

## Supporting information

S1 File Appropriate antibiotic use knowledge dataset. (SAV)
